# Supplementary material for: Municipality Data as a Rapid and Effective Tool to Analyse Spatial and Temporal Variations of All-Cause Mortality by Town District: The Experience in Genoa (Italy)
Source: Int J Environ Res Public Health. 2021 Aug 4;18(16):8250. doi: 10.3390/ijerph18168250 (PMC8394076; doi:10.3390/ijerph18168250)
Supplement: Supplementary file 1 [file ijerph-18-08250-s001.zip › ijerph-1312735-supplementary.pdf]

Table S1. Comparison of some indicators (Genoa vs. Italy).

Indicators	Genoa	Italy
Old Age Index	264.0	184.1
Structural Dependency Index	65.6	56.9
Turnover Index of the Active Population	167.8	139.1
Structure Index of the Active Population	158.9	141.5
Burden Index of Children per Fertile Woman	17.1	18.2
Average Age*	49.2	46.0
Birth Rate (per 10,000 population)*	58.0	68.0
Raw Mortality Rate (per 10,000 population)*	177.0	126.0
Life Expectancy at Birth*	81.4	82.0
Life Expectancy at 65 Years*	19.4	19.9

*Note: * indicates data referring to the entire province of Genoa in 2020 rather than only the municipality.*

The Old Age Index in 2021 for the municipality of Genoa indicates the presence of 264.0 elderly people for every 100 young people, clearly more than in the Italian population. Also the Structural Dependency Index is higher in Genoa than in the whole country: in 2021 in the city there are 65.6 dependent individuals for every 100 who work, while in Italy this figure is 56.9. The Turnover Index of the Active Population in Genoa in 2021 is 167.8; this indicates that the population of working age is very old, even more so than in Italy as a whole (139.1). Similarly, the Structure Index of the Active Population is higher in Genoa (158.9) than in Italy (141.5). The Burden Index of Children per Fertile Woman in Genoa is 17.1, lower than the Italian index (18.2). The Birth Rate, which in Italy in 2020 was already low (annual average 68 births per 10,000 inhabitants), was even lower in the province of Genoa (58). Conversely, the Raw Mortality Rate is higher in Genoa than in Italy (177 versus 126). The Average Age of the Genoa population is greater than that in Italy: 49.2 versus 46 years. Lastly, the Life Expectancy for the population of the province of Genoa is slightly lower than that of Italy, both at birth and at 65 years.

Table S2. Demographic indicators of the 25 districts of Genoa.

DISTRICT NUMBER	DISTRICT NAME	SURFACE (km^2)	RESIDENTS (average 2009-2020)	POPULATION DENSITY (Res/km^2)	% over 65
1	VOLTRI	41.19	12,522	304	23%
2	PRA'	11.96	20,695	1,730	20%
3	PEGLI	24.39	26,596	1,090	23%
4	SESTRI	17.03	45,303	2,660	22%
5	RIVAROLO	12.29	33,863	2,755	19%
6	BOLZANETO	13.74	14,884	1,083	20%
7	PONTEDECIMO	7.21	12,421	1,723	19%
8	CORNIGLIANO	5.20	15,098	2,903	17%
9	SAMPIERDARENA	5.01	44,222	8,827	19%
10	S.TEODORO	2.37	21,964	9,268	21%
11	OREGINA-LAGACCIO	2.75	24,504	8,910	21%
12	PRE-MOLO-MADDALENA	1.54	23,892	15,514	12%
13	CASTELLETTO	1.99	28,142	14,142	22%
14	PORTORIA	1.44	12,598	8,749	23%
15	FOCE	1.19	15,620	13,126	24%
16	S.FRUTTUOSO	2.10	35,031	16,681	24%
17	MARASSI	5.83	40,067	6,873	22%
18	STAGLIENO	6.03	20,943	3,473	21%
19	MOLASSANA	16.62	25,190	1,516	21%
20	STRUPPA	19.14	10,151	530	21%
21	VALLE STURLA	13.52	14,635	1,082	22%
22	S.MARTINO	1.84	15,155	8,236	23%
23	ALBARO	3.00	28,748	9,583	25%
24	QUARTO	4.44	29,648	6,677	24%
25	NERVI-QUINTO-S.ILARIO	18.67	19,203	1,029	24%
	GENOA TOWN	***240.49***	***591,095***	***2,458***	

Table S3. SMRs (males) in the 25 districts of Genoa, 2009-2020, with 90% CIs.

DISTRICT	OBSERVED CASES	EXPECTED CASES	SMR	90% CI
CORNIGLIANO	1207	969.9	124.4	118.7-130.5
PRA'	1759	1418.2	124.0	119.3-129.0
PRE-MOLO-MADDALENA	1364	1193.4	114.3	109.3-119.5
STRUPPA	895	786.7	113.8	107.7-120.2
BOLZANETO	1190	1056.5	112.6	107.4-118.1
RIVAROLO	2564	2288.5	112.0	108.5-115.7
VOLTRI	1126	1045.9	107.7	102.5-113.1
SESTRI	3788	3532.6	107.2	104.4-110.1
OREGINA LAGACCIO	1905	1841.3	103.5	99.6-107.4
PONTEDECIMO	928	910.2	102.0	96.6-107.6
STAGLIENO	1559	1531.1	101.8	97.7-106.2
S.FRUTTUOSO	2920	2877.1	101.5	98.4-104.6
MOLASSANA	1948	1921.8	101.4	97.7-105.2
S.TEODORO	1718	1704	100.8	96.9-104.9
SAMPIERDARENA	3173	3158.3	100.5	97.6-103.4
MARASSI	3135	3205.3	97.8	95.0-100.7
VALLE STURLA	1067	1135.3	94.0	89.4-98.8
CASTELLETTO	2174	2331.4	93.2	90.0-96.6
PEGLI	2157	2334	92.4	89.2-95.7
QUARTO	2409	2660.3	90.6	87.6-93.6
FOCE	1196	1324.9	90.3	86.1-94.7
NERVI-QUINTO-S.ILARIO	1473	1670.2	88.2	84.5-92.1
S.MARTINO	1115	1265.1	88.1	83.9-92.6
PORTORIA	968	1101.3	87.9	83.4-92.7
ALBARO	2159	2633.2	82.0	79.1-84.9
GENOA	***45897***	***45897***	***100***	

Table S4. SMRs (females) in the 25 districts of Genoa, 2009-2020, with 90% CIs.

DISTRICT	OBSERVED CASES	EXPECTED CASES	SMR	90% CI
CORNIGLIANO	1525	1143.2	133.4	127.9-139.1
RIVAROLO	3118	2605.1	119.7	116.2-123.3
STRUPPA	1023	893.2	114.5	108.8-120.6
S.FRUTTUOSO	4392	3873.6	113.4	110.6-116.2
BOLZANETO	1304	1166	111.8	106.9-117.1
CASTELLETTO	3430	3104.2	110.5	107.4-113.6
PRA'	1738	1622.5	107.1	103.0-111.4
PRE-MOLO-MADDALENA	1327	1239.8	107.0	102.3-112.0
OREGINA LAGACCIO	2307	2229.2	103.5	100.0-107.1
SESTRI	4237	4163.8	101.8	99.2-104.4
SAMPIERDARENA	3615	3612.2	100.1	97.4-102.9
PONTEDECIMO	1007	1010.9	99.6	94.6-104.9
VOLTRI	1159	1164.8	99.5	94.8-104.4
PORTORIA	1244	1300.3	95.7	91.3-100.2
QUARTO	2826	2971.8	95.1	92.2-98.1
MOLASSANA	1918	2041.8	93.9	90.5-97.5
S.MARTINO	1401	1494.7	93.7	89.7-97.9
PEGLI	2379	2582.3	92.1	89.1-95.3
S.TEODORO	1711	1860	92.0	88.4-95.7
STAGLIENO	1572	1727.2	91.0	87.3-94.9
MARASSI	3225	3569.1	90.4	87.8-93.0
FOCE	1515	1680.6	90.1	86.4-94.0
VALLE STURLA	1153	1323.6	87.1	83.0-91.4
ALBARO	2953	3414.2	86.5	83.9-89.2
NERVI-QUINTO-S.ILARIO	1697	1982	85.6	82.3-89.1
GENOA	***53776***	***53776***	***100***	

Table S5. Annual SRDs (males) in the 25 districts and the whole city of Genoa.

	2009	2010	2011	2012	2013	2014	2015	2016	2017	2018	2019	2020
BOLZANETO	130.52	111.60	138.22	156.56	163.54	107.31	139.06	121.77	116.76	92.76	110.57	149.52
CASTELLETTO	108.26	122.72	108.69	99.82	103.22	92.98	106.57	100.75	106.65	95.95	92.59	119.54
CORNIGLIANO	141.25	154.76	155.08	139.70	144.58	150.69	140.00	137.03	154.33	103.31	121.96	169.08
FOCE	124.77	113.60	104.80	111.93	90.56	99.57	109.60	89.06	97.93	73.12	111.82	105.69
MARASSI	133.24	111.66	126.54	117.01	101.31	99.67	101.86	109.74	112.81	102.39	99.72	127.22
MOLASSANA	125.89	114.11	119.05	120.17	121.26	104.16	120.68	93.41	109.27	119.96	108.07	123.18
NERVI-QUINTO-S.ILARIO	101.76	112.04	103.61	94.06	98.72	89.42	97.36	94.18	99.19	104.43	112.02	112.62
OREGINA LAGACCIO	119.83	120.13	128.49	117.01	111.60	121.42	130.23	101.07	128.20	117.06	94.18	129.18
PEGLI	115.57	114.82	110.00	112.44	105.94	104.73	97.37	93.18	94.90	87.60	103.41	121.16
PONTEDECIMO	104.80	129.92	123.02	111.54	102.78	107.88	107.51	111.54	117.78	110.28	107.36	150.74
PORTORIA	110.85	88.64	106.85	103.48	110.79	95.89	96.52	77.54	94.01	97.02	102.68	139.69
PRA	145.27	138.65	147.14	134.25	141.86	143.34	146.73	136.96	142.67	129.47	117.71	141.09
PRE-MOLO-MADDALENA	154.13	137.02	122.94	130.06	133.40	117.33	135.79	126.55	133.91	119.32	102.10	146.50
QUARTO	112.72	109.52	108.15	105.07	112.50	85.62	89.24	98.24	98.80	94.03	85.08	120.81
RIVAROLO	116.13	140.97	131.90	141.62	141.41	106.57	123.97	116.90	125.61	123.29	113.70	139.59
S.FRANC.D'ALBARO	97.05	95.65	94.51	95.82	98.08	81.45	96.49	82.42	86.72	80.76	87.48	107.75
S.FRUTTUOSO	121.19	122.75	101.26	112.65	109.25	113.09	113.23	106.70	104.66	112.06	111.00	147.96
S.MARTINO	102.51	114.19	92.00	106.40	100.82	113.98	75.98	96.69	110.78	103.88	82.80	102.29
S.TEODORO	122.38	113.45	117.32	144.70	138.54	137.11	112.86	97.01	97.58	93.66	86.05	122.48
SAMPIERDARENA	122.42	111.94	107.78	118.52	110.77	111.62	116.33	104.04	122.64	93.48	104.44	140.14
SESTRI	136.71	105.16	125.89	135.47	126.87	110.35	110.38	121.89	112.69	121.13	105.82	150.14
STAGLIENO	126.20	106.47	136.08	122.15	96.23	110.30	120.36	126.94	114.39	96.06	100.75	122.83
STRUPPA	117.51	115.28	108.54	130.67	127.24	122.76	115.69	112.47	163.44	167.87	139.96	127.12
VALLE STURLA	118.62	120.77	106.72	99.38	109.87	99.58	89.96	111.77	120.16	98.55	93.63	109.15
VOLTRI	129.34	135.86	131.32	156.30	156.09	113.84	103.54	99.28	101.85	113.26	123.04	126.19
**GENOA**	**121.33**	**115.94**	**116.58**	**117.73**	**115.00**	**106.56**	**110.51**	**105.56**	**112.13**	**104.25**	**102.76**	**130.20**

Table S6. Annual SRDs (females) in the 25 districts and the whole city of Genoa.

	2009	2010	2011	2012	2013	2014	2015	2016	2017	2018	2019	2020
BOLZANETO	82.76	99.24	80.78	100.52	75.42	85.76	95.03	86.85	80.17	82.44	66.08	82.16
CASTELLETTO	82.52	71.16	74.08	80.47	79.06	71.55	69.89	70.70	72.26	75.06	75.50	94.90
CORNIGLIANO	108.05	107.69	93.29	90.27	94.11	93.35	100.63	93.50	92.71	107.71	87.51	110.84
FOCE	65.27	72.59	70.48	64.14	69.05	48.45	74.89	63.40	57.82	64.13	64.46	60.99
MARASSI	67.84	66.29	75.20	73.21	63.48	64.75	67.94	66.22	61.03	62.53	61.30	72.43
MOLASSANA	69.57	69.17	72.37	74.76	62.73	57.47	69.45	70.91	71.54	71.05	65.15	78.48
NERVI-QUINTO-S.ILARIO	60.22	64.67	63.50	60.98	63.59	53.90	58.95	65.93	56.61	63.29	55.68	66.69
OREGINA LAGACCIO	70.14	78.00	71.19	77.95	73.02	61.16	83.73	81.81	80.14	76.54	67.58	80.60
PEGLI	72.83	64.03	65.61	75.37	65.49	61.09	59.95	65.98	62.32	62.91	58.72	72.07
PONTEDECIMO	89.25	74.75	79.08	73.92	70.78	49.27	74.94	72.68	57.19	69.17	73.80	80.26
PORTORIA	71.67	76.15	64.09	73.64	67.94	60.75	59.83	56.96	63.72	59.80	75.48	72.74
PRA	85.81	83.28	86.09	76.07	74.66	77.17	87.43	77.77	69.26	69.10	69.52	92.75
PRE-MOLO-MADDALENA	72.77	86.29	77.38	82.49	73.43	69.68	72.67	82.51	74.58	78.13	88.77	87.21
QUARTO	78.40	80.50	62.20	64.23	66.85	70.66	71.71	57.44	64.36	65.84	61.14	75.98
RIVAROLO	90.09	81.52	87.66	86.02	86.50	75.79	94.89	79.51	77.13	89.65	81.68	105.88
S.FRANC.D'ALBARO	65.67	71.33	61.56	58.16	55.75	62.79	66.37	51.20	57.84	55.65	65.06	60.54
S.FRUTTUOSO	88.59	93.03	90.02	85.89	77.23	77.91	73.43	80.06	72.13	80.40	69.99	103.24
S.MARTINO	72.77	70.09	80.33	74.40	59.08	67.91	58.05	55.50	79.63	72.41	52.09	65.64
S.TEODORO	82.25	75.55	70.89	62.97	77.41	62.98	69.90	53.97	72.12	66.41	49.05	82.03
SAMPIERDARENA	77.82	85.98	74.54	71.29	74.01	74.77	66.57	73.26	72.53	66.99	65.45	78.51
SESTRI	77.63	76.17	78.68	82.57	74.80	71.80	72.89	66.02	72.68	64.66	63.20	83.04
STAGLIENO	72.81	66.66	66.02	67.80	68.43	62.59	66.75	60.45	72.50	61.99	66.71	72.37
STRUPPA	83.95	79.12	99.62	84.02	89.31	54.57	66.62	82.80	78.62	77.57	73.86	100.52
VALLE STURLA	86.23	73.39	63.60	55.50	65.64	52.09	81.65	67.76	59.05	68.34	46.00	65.66
VOLTRI	85.86	68.60	79.90	57.55	78.59	68.49	81.60	58.47	57.57	67.36	74.82	94.34
**GENOA**	**77.66**	**76.93**	**74.97**	**74.24**	**71.61**	**67.37**	**72.72**	**69.21**	**69.33**	**70.36**	**66.53**	**81.44**

Table S7. Total number of deaths registered by the two sources (municipality and ISTAT).

Year	No. of deaths - municipality	No. of deaths - ISTAT	Difference %
2015	8,266	8,376	1.33%
2016	7,941	8,066	1.57%
2017	8,342	8,468	1.51%
2018	8,155	8,226	0.87%
2019	7,998	8,072	0.93%
2020	9,971	10,031	0.60%

Figure S1. Distribution of Genoa population by age and sex, 1st January 2021.

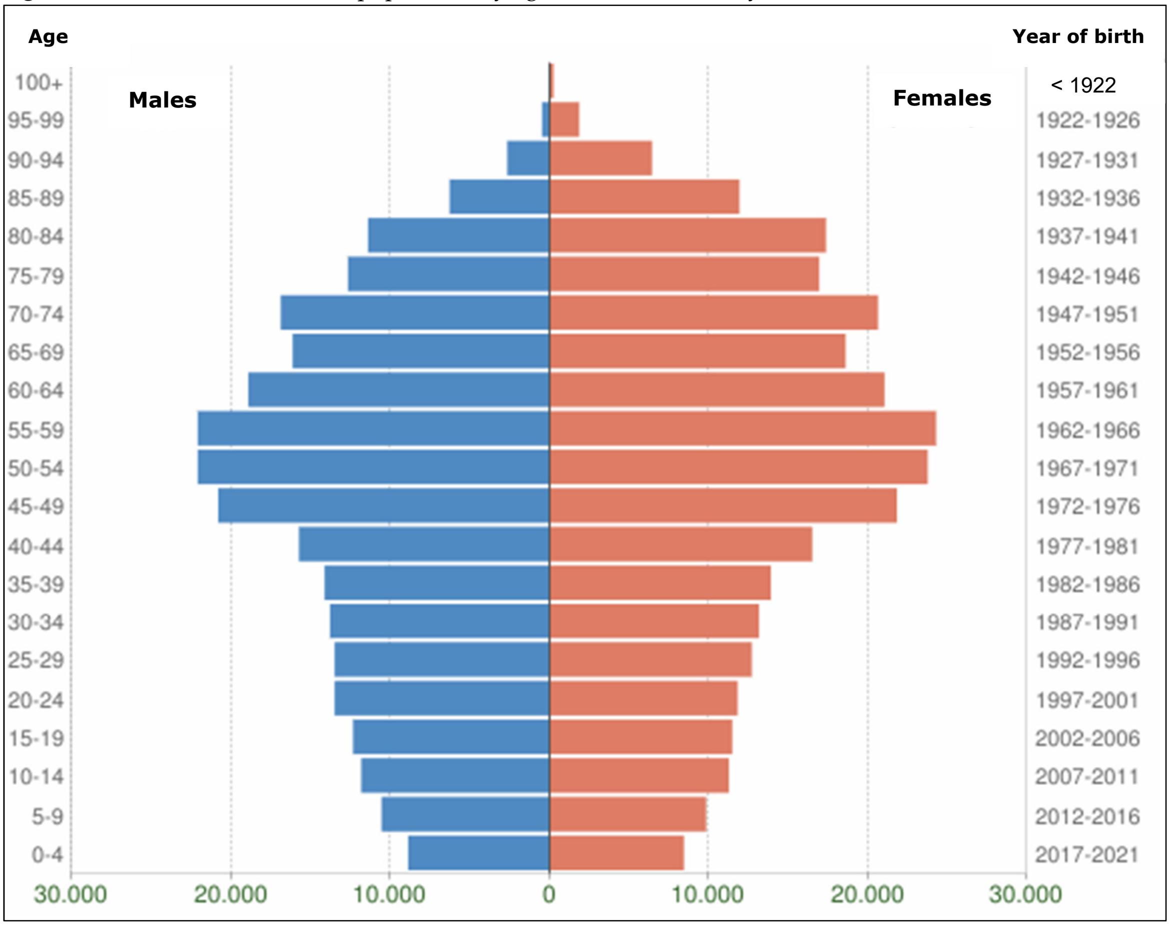

Source: ISTAT – Elaboration: Tuttitalia.it.

Figure S2. Population structure 1st January 2021, Genoa vs. Italy.

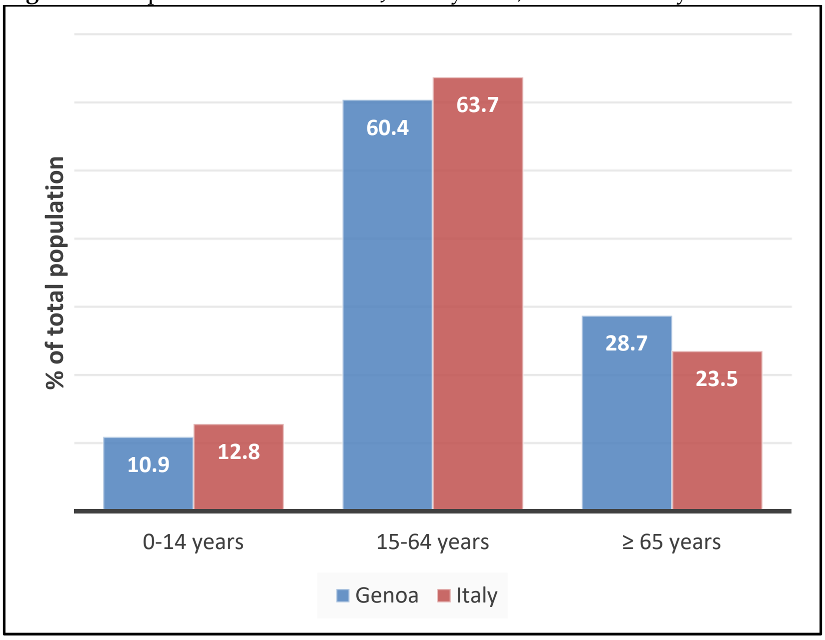

Source: ISTAT.
